# The Borderline Personality Features Scale for Children (BPFSC-11): psychometric properties and convergence with measures of child maltreatment and depression in Vietnamese adolescents

**DOI:** 10.1186/s12888-026-08264-x

**Published:** 2026-06-11

**Authors:** Ha Thu Dinh, Adrian B. Kelly, Huong Thanh Nguyen, Thi Minh Le, Philip R. A. Baker

**Affiliations:** 1 School of Public Health and Social Work, Faculty of Health, Queensland University of Technology, Victoria Park Road, Brisbane, QLD 4059 Australia; 2 Australian Centre for Health Law Research, Queensland University of Technology, Brisbane, QLD 4059 Australia; 3 Faculty of Social and Behavioural Sciences, Hanoi University of Public Health, 1A Duc Thang, Dong Ngac, Hanoi, 100000 Vietnam; 4 School of Psychology and Counselling, Faculty of Health, Queensland University of Technology, Brisbane, QLD 4059 Australia; 5 Department of Public Health Medicine, Faculty of Medicine, Universiti Teknologi MARA, Jalan Hospital, Sungai Buloh Campus, Sungai Buloh, Selangor 47000 Malaysia; 6 Graduate School of Public Health, Yonsei University, 50-1, Yonsei-Ro, Seodaemun-gu, Seoul, 03722 Republic of Korea

**Keywords:** Vietnamese children, Adolescents, Borderline personality, BPFSC-11, Depression, Psychometric properties, Measurement invariance, Abuse and neglect

## Abstract

**Background:**

Borderline personality features (BPF) are developmentally meaningful indicators of emotional, interpersonal, and identity dysregulation in adolescence, which are commonly linked to child maltreatment (CM), depression and suicidal ideation. In Vietnam, where CM and mental health problems among adolescents represent a significant public health concern, no culturally validated tool exists to assess BPF. This study examined the psychometric properties and gender invariance of the Vietnamese version of the Borderline Personality Features Scale for Children (BPFSC-11) and evaluated its convergent validity with depressive symptoms and CM.

**Methods:**

The participants were 2,326 secondary school students in grades 7 and 9 (49.9% female) recruited from 8 randomly selected schools in Northern Vietnam. BPF were measured using the BPFSC-11, while CM was assessed through an adapted Computer-Assisted Maltreatment Inventory scale (CAMI). All instruments were administered in Vietnamese. To examine the factor structure of the BPFSC-11, exploratory factor analysis (EFA) was conducted on a randomly selected half of the dataset, followed by confirmatory factor analysis (CFA) on the remaining half. Measurement invariance across gender was assessed using multigroup CFA (configural, metric, scalar). Convergent validity was examined through correlations between BPF, depressive symptoms and CM.

**Results:**

The BPFSC-11 demonstrated high internal consistency (*α* = 0.87) and a unidimensional factor structure supported by both EFA and CFA. Measurement invariance across genders was established. Female adolescents scored significantly higher on BPF compared to males. Moderate to strong correlations were found between BPF, CM, and depressive symptoms (*r* varies from 0.32 to 0.66, *p* < 0.001), indicating adequate convergent validity.

**Conclusions:**

The Vietnamese BPFSC-11 appears to be a reliable and valid brief measure for assessing BPF in Vietnamese adolescents. Its strong psychometric performance supports its use in research and school-based screening to identify at-risk adolescents, better understand adolescent emotional and interpersonal difficulties, and their associations with CM. Further longitudinal and clinical validation is warranted to examine developmental trajectories and broader applicability.

**Clinical trial number:**

Not applicable.

## Background

Adolescence is a period of rapid changes in affect regulation, identity formation, and interpersonal functioning [1]. During this time, vulnerabilities to mental health and personality-related difficulties can emerge, making adolescence a critical window for early detection of maladaptive emotional and relational patterns. Borderline personality disorder (BPD), formally defined in adulthood as a pervasive pattern of instability in affect, self-image, relationships, and impulse control [2, 3], is increasingly understood to have developmental precursors identifiable in earlier stages of life [4]. These precursors occur along a developmental continuum comprising borderline traits, relatively enduring personality characteristics reflecting emotional sensitivity, interpersonal reactivity, and temperamental vulnerability [5]; and borderline personality features (BPF), which represent more acute, dimensional symptom-like manifestations such as affective instability, identity disturbance, interpersonal sensitivity, fears of abandonment, self-harm tendencies and impulsivity [5]. BPF are not equivalent to a categorical diagnosis of BPD; rather, they represent developmentally meaningful indicators of emerging borderline personality pathology that can be reliably measured in both clinical and community adolescent samples and even older children [6]. Consequently, dimensional assessments of BPF have become central to early detection and prevention frameworks targeting adolescents at risk [6].

The Borderline Personality Features Scale for Children (BPFSC-11) [7] is among the most widely used brief self-report measures of BPF. Developed using item response theory from the original 24-item BPFS-C [8], the BPFSC-11 comprises 11 items capturing key BPF domains, including affective instability, identity problems, negative relationships, and impulsivity/self-harm. Across validation studies conducted in diverse cultural contexts, the instrument has consistently demonstrated strong internal consistency (*α* = 0.72–0.89) and convergent validity [9–15]. Moreover, gender invariance has been confirmed in most studies [9, 10, 13, 15], suggesting that boys and girls interpret and respond to BPFSC-11 items in comparable ways. Findings from the Norwegian study also showed measurement invariance across language background and nativity [10].

Despite this robust literature, the factor structure of the BPFSC-11 has not been consistent across cultures and populations. In the original validation study, Sharp et al. reported a unidimensional structure in a large United States community sample [7], a structure that was subsequently replicated in North America and Western Europe, specifically, France [14], the United States [16], Portugal [13], and Lithuanian [9]. In contrast, evidence from Italy [15], Spain [12], Pakistan [11] and Norway [10] supported a two-factor or bi-factor model, which differentiated emotional/identity instability from interpersonal difficulties/impulsivity. A community-based validation of the BPFS-C among Canadian adolescents also found a two-factor solution [17]. This variability suggests potential cultural and contextual influences on adolescents’ interpretations and responses to BPF items, underscoring the need for local validation before use in new populations.

Beyond measurement considerations, BPF are closely tied to other adolescent mental health outcomes. Child maltreatment (CM), including emotional abuse, neglect, physical and sexual abuse, and witnessing intimate partner violence (IPV) [18–20], is strongly associated with elevated BPF, both through direct psychological impacts and through disruptions to emotion regulation, identity formation, and interpersonal functioning [5, 21–27]. These patterns align with Linehan’s invalidating environment framework, which posits that chronic emotional invalidation undermines self-regulation and contributes to borderline traits [24]. BPF also show robust associations with depression, with shared mechanisms such as emotion dysregulation and internalising distress well documented across community and clinical samples [2, 28]. Disturbances in the sense of self, such as feelings of fragmentation, abandonment, or emotional unsafety, are similarly interwoven with suicidal ideation [24, 29, 30] and are frequent among adolescents exposed to maltreatment [30]. Evidence also suggests that female adolescents experience higher levels of both depressive symptoms and BPF than males [2, 5, 31–33]. These well-established associations provide a strong rationale for examining the convergent validity of the BPFSC-11 with measures of CM and depressive symptoms.

The relevance of these associations is heightened in Vietnam, where adolescent mental health burdens are substantial. Vietnam has approximately 16 million adolescents aged 10–19 years (15.9% of the population) [34], and national surveys consistently report rising emotional distress. The Survey Assessment of Vietnamese Youth (SAVY I–II) documented increases in suicidal ideation (from 3.4% to 4.1%) [35], while the 2019 Global School-based Student Health Survey (GSHS) found that 31.7% of adolescents reported depressive symptoms and 11% reported suicidal ideation [36], rates exceeding those of many neighbouring Southeast Asian countries [37]. Non-suicidal self-injury is also highly prevalent, with 43.9% of adolescents reporting at least one incident in the past 12 months and over 25% reporting multiple episodes [38].

CM is similarly widespread [39–42]. A community-based study in Northern Vietnam showed that one in two adolescents had experienced at least one form of CM in the past year [39]. Numerous studies have established strong links between CM, depression and suicidality among Vietnamese adolescents [40–42]. The relationship between CM and depression has been well documented using psychometrically validated tools such as the Center for Epidemiological Survey Depression Scale (CES-D) [43], which has been widely accepted and utilised in research and screening in Vietnam [44]. In contrast, BPF are far less understood in Vietnam, largely due to the absence of culturally validated assessment tools for adolescents. This limits the field’s ability to characterise borderline-related traits, investigate their developmental determinants, or understand their role in the psychological impact of CM.

The absence of a validated tool also constrains school-based identification and broader public health monitoring of borderline-related difficulties. Establishing the psychometric properties of a brief, feasible instrument like the BPFSC-11 is therefore critical. This study aims to validate the Vietnamese version of the BPFSC-11 in a large school-based sample of adolescents. Specifically, we examine [1] internal consistency [2], factor structure via exploratory and confirmatory factor analyses [3], measurement invariance across gender, and [4] convergent validity with depressive symptoms and multiple forms of CM.

Based on prior BPFSC-11 studies and the documented links among BPF, CM, and depression, we hypothesised that: [1] the Vietnamese BPFSC-11 would demonstrate high internal consistency and a sound factor structure consistent with earlier work; [2] the scale would exhibit measurement invariance across gender, with female adolescents reporting higher BPF scores; and [3] BPFSC-11 scores would show moderate to strong correlations with depressive symptoms and CM.

## Methods

### Participants

The analysis sample comprised 2,326 students (49.9% female) in grades 7 (50.2%, mean age 12 years) and 9 (49.8%, mean age 14 years). Among the full sample, 46.3% lived in rural areas while the other 53.7% lived in a town or city, and almost all (97.2%) of the students lived with their families. The study was conducted in Bac Giang, a semi-rural yet fast-urbanising province in Northern Vietnam, with a population of 1.92 million [45]. The province’s economic growth was ranked third at the national level, with the poverty rate declining from 90% in 1995 to 9.5% in 2018 and to 1.5% in 2023 [45]. In 2023, the province’s average monthly income per capita was USD 194.98, closely aligning with the national average of USD 208.4 [45]. Other key development and health indicators, including literacy, life expectancy, infant health, and insurance coverage, similarly mirrored national patterns [45].

### Procedure

This research was part of a larger project, conducted by researchers from Vietnam and Switzerland from 2022 to 2025, which examines the prevalence of CM and its impact on the mental health of adolescents in both countries. A multi-stage sampling approach was employed. In the first stage, two districts, one urban and one rural, were randomly selected from a total of 10 districts. In the second phase, four schools were randomly chosen from among all schools within each district using a random number generator. In the third stage, the survey team randomly selected 4–5 classes from grade 7th and 4–5 classes from grade 9th, depending on the class size. The sampling frame comprised 2,661 students across 66 classes from 8 schools. After the consent process, 1.1% of the students were excluded due to a lack of parental consent or student assent, resulting in 2,632 participants eligible to take part in the survey. Data were collected through a self-administered classroom-based survey using tablets pre-installed with KoboToolbox software [46]. Among those eligible, 2,356 (89.5%) participated and completed the survey. The reason was either that some students did not appear for the survey or that they left early for another session, given that the survey lasted for approximately 45–60 min.

### Tools translation and adaptation

The questionnaire was initially co-developed in English by experts involved in a collaborative research partnership between Vietnam and Switzerland, with the objective of producing a harmonised instrument for cross-country comparisons. After consensus was reached on the final English version, the instrument was translated into Vietnamese and German through a parallel translation process in which three independent translators generated separate versions for each target language. Across both study sites, the translated instruments underwent a multi-phase, theory-informed process of cultural and linguistic adaptation to ensure their appropriateness for adolescents in each setting. In the first stage, the parallel translations were subjected to expert review by the research team, who evaluated item relevance, developmental appropriateness, cultural resonance, and conceptual equivalence across contexts. During this expert review, translation inconsistencies were also identified and resolved. For example, item BP11 of the BPFSC-11 *“Lots of times*,* my friends and I are really mean to each other”*, had been incorrectly translated by one translator as *“Lots of times*,* my friends and I mean a lot to each other”*, which conveys the opposite meaning. This discrepancy was detected during the expert review process and corrected prior to testing with adolescents. In the second stage, two focus group discussions were conducted with adolescents to assess clarity of item wording, comprehension, and underlying interpretive processes. Feedback from these sessions informed targeted refinements to both phrasing and response options. For instance, an item on the neglect subscale of CM, originally worded as *“There were times in my life*,* my clothes didn’t fit me”*, was interpreted by adolescents as referring to the popular fashion trend of wearing oversized clothing rather than to neglect. The item was therefore revised to *“There were times in my life when I had to wear clothes that didn’t fit me because I didn’t have anything else”* to improve conceptual clarity. In the third stage, appropriateness and relevance for Vietnamese adolescents were further assessed through a pilot survey of 241 adolescents from the same province where the main study was conducted. Insights from the pilot study guided additional refinements and cultural adaptations to ensure fidelity and interpretability across both contexts. This iterative, year-long development process was designed with the aim of ensuring the content validity, cross-cultural relevance, and ecological appropriateness of the instruments for use in both Vietnam and Switzerland.

### Data quality check

After data cleaning to remove low-quality responses, the final analytic sample comprised 2,326 students. Records were excluded if they exhibited [1] uniform response patterns on Likert-type items that were logically inconsistent, particularly given the presence of reverse-scored items, and [2] exceptionally short completion times, defined as fewer than 10 min for the full survey.

### Measures

#### Borderline personality features

These were measured using the Borderline Personality Features Scale for Children (BPFSC-11) [7]. This 11-item version has been recommended for measuring BPF in children (aged 9–18 years) and has shown psychometric utility in diverse settings [7, 9, 13–16]. However, its construct validity in the Vietnamese context is unclear. The items were scored on a 5-point Likert scale ranging from *1 “Not at all true”* to *5 “Always true”* (e.g., *“I go back and forth between different feelings*,* like being mad or sad or happy”; “I want to let some people know how much they’ve hurt me”*).

#### Child depression

This was measured using the Vietnamese version of the Center for Epidemiological Survey Depression Scale (CESDR-V) [47], a previously validated 20-item instrument. The CESDR-V showed a single latent dimension, measurement invariance across gender, convergent validity with the DASS-21 Depression scale, and excellent internal consistency, with Cronbach’s alpha 0.92 in the original validation study and 0.91 in the current sample [48]. Responses were collected on a 5-point scale from *1 “Not at all or less than one day in the past week”* to *5 “Nearly every day for two weeks”*.

#### Child maltreatment

CM was assessed using the adapted Computer-Assisted Maltreatment Inventory scale (CAMI) [49]. The measure covers five subtypes of CM: physical abuse (5 items; e.g., *“At any time in your life*,* did either of your parents ever spank*,* hit or kick you?”*), sexual abuse (4 items; e.g., “*At any time in your life*,* did anyone against your will kiss*,* touch*,* or fondle your body (outside your clothing*,* under your clothing*,* or when undressed) in a sexual way or forced you to touch or kiss them?”*), emotional abuse (26 items; e.g., *“There was a time in my life (now or in the past) when at least one of my parents cursed or swore at me”*), neglect (29 items; e.g., *“In the past and present*,* there were times when my parents did not care whether I go to school”*) and witnessing IPV (4 items; e.g., *“At any time in your life*,* did you witness a parent getting pushed*,* shook*,* slapped*,* or kicked by another parent or by their boyfriend or girlfriend? Or*,* did you witness a parent doing these things with her/his partner?”*). Responses to items on physical abuse, sexual abuse and witnessing intimate partner violence were coded dichotomously as *“Yes”* or *“No”*. In contrast, responses on emotional abuse and neglect were scored on a 5-point Likert scale, ranging from *1 “Strongly disagree”* to *5 “Strongly agree”*. The categories *“Strongly disagree”* and *“Disagree”* were combined and recoded as 0. This recoding was based on the rationale that both *“Strongly disagree”* and *“Disagree”* indicate an absence of perceived emotional abuse or neglect. Conversely, the categories *“Neutral”*, *“Agree”*, and *“Strongly agree”* were recoded as 1, 2, and 3, respectively, to reflect varying levels of perceived emotional abuse or neglect. The maximum possible score for each subtype of CM was 5 for physical abuse, 4 for sexual abuse, 4 for intimate partner violence, 78 for emotional abuse, and 87 for neglect. The overall CM score was calculated by summing the scores of all five subtypes, yielding a maximum score of 178. The adapted Vietnamese CAMI demonstrated acceptable to excellent internal consistency across subscales in the current sample (emotional abuse *α* = 0.92; neglect *α* = 0.90; sexual abuse *α* = 0.70; physical abuse *α* = 0.54; witnessing IPV *α* = 0.66), and its factor structure was supported by both EFA and CFA [48].

### Ethical considerations

The broader Vietnam CM study received ethical approval from the Research Ethics Committee of Hanoi University of Public Health, Vietnam (Decision number 321/2022/YTCC-HD3, issued on 11th July 2022). Participation in the survey was limited to adolescents who provided active assent and whose parents gave written informed consent. On-site psychological support for adolescents was available to participants throughout the data collection period. This study is part of a doctoral thesis conducted in Australia and was approved by the University Human Research Ethics Committee (UHREC) of Queensland University of Technology, Australia, under a waiver of consent (Approval number 9411, issued on 4th February 2025). All principles expressed in the Helsinki Declaration and the National Statement on Ethical Conduct in Human Research of the Australian Research Council have been met.

### Statistical analysis

In Step 1, coefficient alpha was used to evaluate the internal consistency of the BPFSC-11 scale. In Step 2, the dataset was randomly split into two equal subsamples of 1,136 cases (Subsample A and Subsample B) using a random number generator to allow for independent validation of the scale’s structure. Exploratory factor analysis (EFA) was conducted on Subsample A to explore latent dimensions, followed by Confirmatory factor analysis (CFA) on Subsample B to confirm the model fit (Step three). This approach was employed because of inconsistent evidence on the factor structure of the BPFSC-11 worldwide, and there is limited understanding of how this scale would function in an Asian context, such as Vietnam. EFA was conducted using Principal Axis Factoring, an extraction method recommended for identifying underlying latent constructs. Promax oblique rotation was applied, given the conceptual expectation that BPF would be correlated. For item retention, a minimum factor loading threshold of 0.40 was used [50], together with consideration of Cronbach’s alpha when an item is deleted. The number of factors was evaluated using multiple criteria, including inspection of the component matrix, eigenvalues greater than 1, Scree plots, and total variance explained [50]. The assessment of model fit was guided by the decision tree proposed by Mai et al. [51], which argued that model fit indices are inherently context-sensitive and that a universal evaluation approach remains elusive. Instead, they advocated for a “tailored-fit model evaluation strategy” that considers model novelty and focuses on measurement or structural models and sample size. For our large sample size, the recommended best-performing indicator of CFA’s model fit was a CFI close to 0.971 [51]. Additionally, it is also suggested that the CFI plus SRMR should be close to 0.971 [51].

In Step 4, measurement invariance across gender was evaluated using multi-group CFA following the standard sequential procedure (configural, metric, scalar). From this step onward, all analyses were conducted using the full sample. Model fit was assessed using the same criteria in Step 3 [51]. To determine whether a more restricted model demonstrated meaningfully worse fit than its less restricted counterpart, changes in model-fit indices (ΔCFI, ΔRMSEA, ΔSRMR) were examined. Consistent with simulation-based recommendations, invariance was considered supported when ΔCFI < 0.010, ΔRMSEA < 0.015, and ΔSRMR < 0.030 for metric invariance and < 0.010 for scalar invariance [52]. In Step 5, the psychometric performance of the BPFSC-11 across genders was further examined using independent-samples *t*-tests.

In the final step, convergent validity of the BPFSC-11 was assessed by examining the correlations of BPF scores with depressive symptoms and CM using Pearson’s correlation. A correlation coefficient (*r*) of around 0.3 indicates a moderate correlation, and a coefficient of around 0.5 indicates a strong correlation [50]. SPSS 30.0 [53] was used for data cleaning and analysis. Jamovi 2.6.44 [54] was employed to perform CFA.

## Results

The analysis consisted of 2,326 students (49.9% female) and participants were evenly distributed across grades 7 (50.2%, mean age 12 years) and 9 (49.8%, mean age 14 years) (Table 1).

**Table 1:** Participants’ characteristics

Characteristics		*n* = 2,326	
Grade		7th	1,168 (50.2%)
		9th	1,158 (49.8%)
Age		12	1,160 (49.9%)
		14	1,138 (48.9%)
		11, 13, 15, 16	25 (1.2%)
Gender		Male	1,132 (48.7%)
		Female	1,160 (49.9%)
		Non-binary	34 (1.5%)
Living area		Small village	1,076 (46.3%)
		Town or city	1,250 (53.7%)
Living circumstance		Live with parents or relatives	2,261 (97.2%)
		Do not live in a family	65 (2.8%)
Mother still alive			2,302 (99%)
Father still alive			2,238 (96.2%)
Depressive symptoms score			Mean 32.4; SD 13.2
Physical abuse			Mean 1.2; SD 1.0
Sexual abuse			Mean 0.2; SD 0.6
Emotional abuse			Mean 7.9; SD 9.7
Neglect			Mean 18.1; SD 9.7
Witnessing intimate partner violence			Mean 0.3; SD 0.7
Child maltreatment			Mean 27.7; SD 16.2

Mean score of the total BPFSC-11 was 27.60, 95% CI (27.2; 28.0), SD 9.58. Mean scores for items ranged from 2.04 for BP5 (*“I’m careless with things that are important to me”*) to 3.20 for BP3 (*“My feelings are very strong. For instance*,* when I get mad*,* I get really really mad. When I get happy*,* I get really really happy”*) (Table 2). In Step 1, the Cronbach’s alpha of the BPFSC-11 was 0.87, indicating good internal consistency [50].

**Table 2:** Borderline Personality Features Scale-11: Cronbach’s alpha, factor loadings, descriptive statistics and model fit indices

Items	Content	Cronbach’s Alpha	Factor loadings	Mean	SD
		**0.87**			
BP1.	I feel very lonely.		0.62	2.06	1.15
BP2.	I want to let some people know how much they’ve hurt me.		0.65	2.46	1.35
BP3.	My feelings are very strong. For instance, when I get mad, I get really really mad. When I get happy, I get really really happy.		0.60	3.20	1.39
BP4.	I feel that there is something important missing about me, but I don’t know what it is.		0.63	2.80	1.38
BP5.	I’m careless with things that are important to me.		0.49	2.04	1.22
BP6.	People who were close to me have let me down.		0.63	2.10	1.21
BP7.	I go back and forth between different feelings, like being mad or sad or happy.		0.74	2.51	1.40
BP8.	I get into trouble because I do things without thinking.		0.63	2.38	1.28
BP9.	I worry that people I care about will leave and not come back.		0.61	2.72	1.59
BP10.	How I feel about myself changes a lot.		0.56	2.89	1.33
BP11.	Lots of times, my friends and I are really mean to each other.		0.55	2.42	1.31
**BPF total score**				**27.60**	**9.58**

Note: χ² [44] = 148, *p* < 0.001, Comparative fit index (CFI) = 0.974,

Standardised root mean square residual (SRMR) = 0.025, Tucker–Lewis index (TLI) = 0.967, Root mean square error of approximation (RMSEA) = 0.045

In Step 2, descriptive analysis showed no meaningful differences between Subsample A and Subsample B. The findings of the EFA on Subsample A indicated that all conditions for running factor analysis were satisfied (Kaiser–Meyer–Olkin measure (KMO) > 0.9, Bartlett’s test of sphericity had a *p* value < 0.001) [50]. All items of the BPFSC-11 loaded onto a single factor, with factor loadings ranging from 0.49 for BP5 (*“I’m careless with things that are important to me”)* to 0.74 for BP7 *(“I go back and forth between different feelings*,* like being mad or sad or happy”*) (Table 2), and item–total correlation varying from 0.45 to 0.68, indicating good factor structure. Cronbach’s alpha would decrease if any item were deleted, demonstrating that all 11 items of the scale were worth retaining. All BPF items were significantly intercorrelated, with correlations ranging from 0.23 to 0.49 (all *p* < 0.001), supporting the suitability of using a Promax-rotation solution. The unidimensional model explained 43.1% of the variability in the BPF. Overall, findings from EFA indicated that the BPFSC-11 had good construct validity.

In Step 3, a CFA was performed on Subsample B to evaluate the single-factor structure of the BPFSC-11 identified in the EFA. Model fit indices (Table 2) indicated that the unidimensional model of the BPFSC-11 had a good model fit (χ²[44] = 148, *p* < 0.001,* CFI = 0.974*,* SRMR = 0.025*,* TLI = 0.967*,* RMSEA = 0.045)* [50, 51]. This finding confirms the single latent dimension of the BPFSC-11, meaning that all 11 items are strongly intercorrelated and measure a unified underlying construct.

In Step 4, measurement invariance across gender was evaluated using multi-group CFA of the single-factor BPFSC-11 model (Table 3). From this step onward, the full sample was used. The configural model demonstrated good fit (CFI = 0.960, TLI = 0.950, RMSEA = 0.052, SRMR = 0.030) [51]. Introducing equality constraints on factor loadings (metric model) resulted in only slight changes in model fit (ΔCFI = 0.005; ΔRMSEA = 0.001; ΔSRMR = 0.011), supporting metric invariance [52]. Adding intercept constraints (scalar model) further produced similarly minimal differences (ΔCFI = 0.009; ΔRMSEA = 0.002; ΔSRMR = 0.004), indicating scalar invariance [52]. Taken together, the findings confirmed that the BPFSC-11 demonstrated measurement invariance across gender.

**Table 3:** Model fit indices for multi-group models testing measurement invariance across gender of the BPFSC-11

	χ²	df	*p*	CFI	TLI	RMSEA	SRMR	ΔCFI	ΔRMSEA	Δ SRMR
Configural	363	88	< 0.001	0.960	0.950	0.052	0.030	*N*/A	*N*/A	*N*/A
Metric	409	98	< 0.001	0.955	0.950	0.053	0.041	0.005	0.001	0.011
Scalar	485	108	< 0.001	0.946	0.945	0.055	0.045	0.009	0.002	0.004

In Step 5, the psychometric properties of the BPFSC-11 were further evaluated by comparing scores across genders. Female students scored significantly higher on the BPF than their male counterparts, with mean BPF scores of 30.30 and 24.80, respectively (mean difference 5.56, 95% *CI* (4.81–6.31), *p* < 0.001) (Table 4). Item-level analyses showed consistent gender differences across all BPF items, with mean differences ranging from 0.22 (BP11 *“Lots of times*,* my friends and I are really mean to each other”*) to 0.73 (BP2 *“I want to let some people know how much they’ve hurt me”*) (*p* < 0.001) (Table 4). Similarly, female adolescents reported greater levels of depressive symptoms than male adolescents did, with mean scores of 34.3 and 29.8, respectively (mean difference 4.5, 95% *CI* (3.5–5.5), *p* < 0.001). These findings support the universal gender disparity of BPF and depression in adolescents and confirm the psychometric functioning of the BPFSC-11.

**Table 4:** Borderline personality features by gender

	Female		Male		Mean difference	95% CI
	Mean	SD	Mean	SD		
BP1	2.34	1.15	1.78	1.08	0.56**	0.47–0.65
BP2	2.82	1.33	2.09	1.28	0.73**	0.62–0.84
BP3	3.49	1.30	2.91	1.42	0.58**	0.47–0.70
BP4	3.01	1.32	2.58	1.41	0.43**	0.31–0.54
BP5	2.21	1.24	1.88	1.18	0.33**	0.23–0.43
BP6	2.39	1.27	1.80	1.07	0.60**	0.50–0.69
BP7	2.83	1.40	2.18	1.33	0.65**	0.54–0.76
BP8	2.61	1.28	2.14	1.24	0.47**	0.37–0.58
BP9	3.02	1.58	2.42	1.54	0.60**	0.47–0.73
BP10	3.08	1.25	2.68	1.37	0.40**	0.30–0.51
BP11	2.53	1.29	2.31	1.31	0.22**	0.11–0.33
**Total BPF**	**30.30**	**9.39**	**24.80**	**8.95**	5.56**	**4.81–6.31**

** *p* < 0.001

In the final step, the CESDR-V and the measure of CM were employed to assess the convergent validity of the BPFSC-11. The correlation matrix (Table 5) indicated that these three constructs were interrelated, with associations more pronounced among female adolescents compared to males. Specifically, the BPF showed a strong correlation with depression (*r* = 0.66 for females and 0.57 for males, *p* < 0.001) and a moderately strong correlation with CM (*r* = 0.51 for females and 0.32 for males, *p* < 0.001). Among five subtypes of CM, the BPF was most closely linked to emotional abuse (*r* = 0.55 for females and 0.47 for males, *p* < 0.001), followed by physical abuse (*r* = 0.28 for females and 0.26 for males, *p* < 0.001), witnessing intimate partner violence (IPV) (*r* = 0.26 for females and 0.18 for males, *p* < 0.001), and finally sexual abuse (*r* = 0.16 for females, *p* < 0.001 compared with *r* = 0.07 for males, *p* < 0.05). Neglect was moderately associated with BPF among female adolescents (*r* = 0.25, *p* < 0.001), whereas no significant relationship was found for males (*r* = 0.06, *p* > 0.05).

**Table 5:** Correlations between borderline personality features, depressive symptoms and child maltreatment for each gender

	1	2	3	4	5	6	7	8
1. BPF	1	0.66^**^	0.28^**^	0.16^**^	0.55^**^	0.25^**^	0.26^**^	0.51^**^
2. Depression	0.57^**^	1	0.26^**^	0.20^**^	0.56^**^	0.31^**^	0.28^**^	0.55^**^
3. Physical abuse	0.26^*^	0.27^**^	1	0.19^**^	0.45^**^	0.24^**^	0.25^**^	0.48^**^
4. Sexual abuse	0.07^*^	0.15^**^	0.20^**^	1	0.25^**^	0.21^**^	0.30^**^	0.33^**^
5. Emotional abuse	0.47^**^	0.50^**^	0.46^**^	0.20^**^	1	0.38^**^	0.35^**^	0.87^**^
6. Neglect	0.06	0.16^**^	0.19^**^	0.14^**^	0.21^**^	1	0.18^**^	0.78^**^
7. Witnessing IPV	0.18^**^	0.17^**^	0.23^**^	0.38^**^	0.31^**^	0.10^*^	1	0.38^**^
8. Child maltreatment	0.32^**^	0.41^**^	0.47^**^	0.27^**^	0.74^**^	0.81^**^	0.30^**^	1

Note: Values in the upper triangle represent correlations for females, while those in the lower triangle represent correlations for males

* *p* < 0.05; ** *p* < 0.001

Depressive symptoms were also strongly associated with CM (*r* = 0.55 for females and 0.41 for males, *p* < 0.001). Depressive symptom scores correlated with all subtypes of CM across both sexes (*p* < 0.001). Again, emotional abuse appeared to be the strongest predictor of depression compared with other types of CM (*r* = 0.56 for females and 0.50 for males, *p* < 0.001), followed by neglect (*r* = 0.31 for females and 0.16 for males, *p* < 0.001), witnessing IPV (*r* = 0.28 for females and 0.17 for males, *p* < 0.001), physical abuse (*r* = 0.26 for females and 0.27 for males, *p* < 0.001), and finally sexual abuse (*r* = 0.20 for females and 0.15 for males, *p* < 0.001).

## Discussion

### Summary of main findings

This study provides the first validation of the BPFSC-11 in Vietnam, demonstrating strong internal consistency, a robust unidimensional structure supported by both EFA and CFA, and clear measurement invariance across gender. The scale showed meaningful convergence with depressive symptoms and multiple forms of CM, with particularly strong associations for emotional abuse. Together, these findings support the reliability and construct validity of the Vietnamese BPFSC-11 in a large, school-attending adolescent sample from a semi-rural, rapidly developing Vietnamese province.

### Factor structure and psychometric interpretation

A single-factor model showed excellent fit in both exploratory and confirmatory analyses, with loadings consistent with prior unidimensional validations in Western countries (the United States, France, Italy, Portugal, and Lithuania) [7, 9, 13, 14, 16]. This pattern suggests that, in this Vietnamese sample, emotional, identity-related, interpersonal, and impulsivity-related difficulties form a coherent latent construct [14].

However, international research has increasingly reported two-factor or bifactor structures, particularly in Italy [15], Spain [12], Pakistan [11], Canada [17], and Norway [10], where emotional/identity instability and interpersonal/impulsivity domains separate more distinctly. These discrepancies point to potential cultural or developmental influences on how adolescents interpret specific BPF items. Although the current data supported a unidimensional model, future work in Vietnam, particularly with clinical or trauma-exposed samples, should compare alternative structural models to evaluate whether multidimensionality emerges under different symptom conditions.

### Convergent validity and construct distinctiveness

The BPFSC-11 showed strong correlations with depressive symptoms and moderate to strong associations with global CM and all CM subtypes. These patterns aligned with established theoretical links between these constructs [2, 5] and are consistent with prior empirical evidence. Across studies, children exposed to CM consistently reported higher levels of BPF [21–23, 25–27] and elevated depressive symptoms [20, 40–42, 55–57]. Moreover, BPF often co-occurs with depression, as documented in both diagnostic frameworks and contemporary research [2, 28].

Emotional abuse was the strongest correlate of depression and BPF compared to the other four subtypes of CM. This finding aligns with evidence from a meta-analysis and a study conducted in Vietnam, which suggested that emotional abuse may have a stronger association with depression than other forms of CM [40, 55]. The strong link between emotional abuse and the BPF can also be explained by Linehan’s theoretical framework on the psychopathology of BPD [24], which posits that individuals with borderline personality disorder exhibit heightened sensitivity to emotional stimuli. According to this theory, when a person’s emotional experiences are consistently invalidated through dismissal, trivialization, or punishment by caregivers or significant others, it can manifest in behaviours such as ignoring the individual’s emotions, criticising them for expressing feelings, or responding unpredictably to their emotional displays or overcontrolling their life. Over time, such an invalidating environment teaches individuals that their emotions are inappropriate or unacceptable, thereby undermining their ability to trust and regulate their own emotional experiences, which eventually results in emotion dysregulation or emotion instability, one of the main characteristics of BPD. This finding supports the use of BPF as a promising predictor of the long-term impact of CM.

Nonetheless, the magnitude of the correlation between BPF and depressive symptoms indicated substantial shared variance and raised concerns about construct distinctiveness. These findings suggest that the scale may partly capture general distress or depressive affect in non-clinical adolescent samples, particularly for items reflecting intense negative emotions or loneliness. While such sensitivity may be beneficial for identifying adolescents with elevated internalising problems in school-based screening, particularly in the context of trauma, it may limit the specificity of the BPFSC-11 as a robust screening tool for BPD in clinical contexts. Future research should more rigorously evaluate the scale’s discriminant validity.

### Gender differences and measurement invariance

Both the BPFSC-11 and the CESDR-V were found to be sensitive to gender differences, with female adolescents exhibiting higher scores for BPF and depressive symptoms than male adolescents did. Given that the BPFSC-11 demonstrated robust measurement invariance across gender, these observed differences are unlikely to reflect measurement artefacts or differential item functioning. This pattern aligns with the existing literature, which consistently demonstrates that female adolescents are at a heightened risk for both depression and BPF compared to their male counterparts [2, 5, 31–33]. In Vietnam, male students’ lower BPF scores may also partly reflect Confucian-influenced masculinity norms, emphasising toughness and emotional restraint [58], which might discourage boys from acknowledging or reporting internalising distress. Such traditional expectations of male emotional suppression contrast with the greater social acceptance of emotional expression among girls, likely contributing to higher self-reported BPF scores in female students. Likewise, the associations between CM and BPF were stronger among female students, which may also be partly due to this cultural attribute. Understanding gender-based differentiation is therefore valuable for informing culturally gender-sensitive research and intervention practice.

### Cultural considerations in the Vietnamese context

Cross‑cultural interpretation of BPF requires attention to culturally embedded norms surrounding emotional expression, family hierarchy, conflict management and disciplinary practices. In Vietnam and other collectivist contexts affected by longstanding disciplinary traditions grounded in Confucian and nationalist ideologies, expectations of filial piety, emotional restraint, and interpersonal harmony shape how adolescents perceive and report affective instability, interpersonal conflict or parental behaviours. Practices that are framed as abusive in Western contexts, such as harsh verbal reprimand or corporal punishment, may be interpreted as normative discipline by Vietnamese adolescents [59, 60], affecting their endorsement of specific CM items and their associations with BPF. Meanwhile, norms emphasising family cohesion and *“saving face”* can further discourage self-report of distress or family adversities [61, 62].

These cultural norms may also shape both the overall mean BPFSC-11 scores and the item functioning of specific items. For example, item BP11 (*“Lots of times*,* my friends and I are really mean to each other”)* has demonstrated weak factor loadings in several Western community samples, including those from Norway, France, and the United States [10, 14, 16]. In contrast, it showed an acceptable loading of approximately 0.50 in this Vietnamese sample, consistent with findings from Portugal [13], and substantially higher loadings exceeding 0.70 in community adolescent samples from Pakistan and Lithuania [9, 11], as well as in a mixed adolescent sample from Spain [12]. Similarly, the total mean BPF score observed in this Vietnamese sample appears broadly comparable to that reported in Lithuania [9] and somewhat lower than scores documented in Norway and Italy [10, 15]. These cross-national differences collectively suggest that cultural norms surrounding peer interaction, interpersonal conflict, and the expression of emotional distress might shape adolescents’ interpretations of BPF-related behaviours.

In the Vietnamese context, peer interactions are embedded within collectivist norms that prioritise interpersonal harmony, face-saving, and sensitivity to relational boundaries, such that playful teasing is largely normalised within close friendships where mutual familiarity affords greater tolerance for minor conversational missteps [63]. Among individuals regarded as *“outsiders”*, however, interactional norms become more restrained, rendering teasing socially risky because speakers are expected to exercise heightened caution to avoid threatening face or disrupting social harmony [63]. This might help explain why item 11 has a considerable loading in this Vietnamese sample.

Taken together, these cultural considerations emphasise that sound psychometric properties and gender invariance do not guarantee cross-national invariance in meaning, thresholds for recognising emotional distress, peer interactions, abuse or parenting practices. Interpretation of BPF scores, depression, and maltreatment in Vietnam must therefore remain culturally informed, and cross-cultural comparisons should be approached with caution until broader invariance testing is conducted.

### Practical implications for research and school-based screening

The brevity of the BPFSC-11 scale makes it practical for use in school settings, and it shows promise for broader application in community contexts, particularly in low-resource contexts. This is particularly important given that BPF during adolescence are still developing and may be malleable, making this developmental stage a critical window for the early identification of high-risk individuals and timely intervention [64], key steps in preventing the progression of BPF into more severe personality disorders. However, given the cross-sectional design, the substantial overlap with depression, and the absence of clinical validation, the BPFSC-11 should not yet be considered a diagnostic tool for borderline pathology. Instead, it may serve as part of a broader school-based screening approach that includes depressive symptoms, behavioural indicators, and contextual risk factors.

Given that suicide remains one of the leading causes of death among adolescents, peaking between ages 15 and 19 [65], and is closely linked to BPF [2, 24, 29], a tool like BPFSC-11, which can assess BPF from age 9 onward, may be crucial for the early identification of adolescents at risk for suicidal ideation or behaviour. Integrating this scale into routine school-based mental health surveillance, alongside widely used measures of depressive symptoms, may offer a more holistic approach to monitoring students’ psychological well-being, particularly in light of rising rates of suicidal thoughfts and attempts among youth [37].

The demonstrated convergent validity of the BPFSC-11 with measures of CM observed in this study indicates that the scale is sensitive to patterns of emotional and interpersonal difficulties commonly observed among adolescents with histories of adversity. This suggests that the instrument may be useful for identifying adolescents who exhibit elevated BPF in contexts where maltreatment is a concern. The development of reliable, valid, and resource-efficient screening tools is essential for the timely detection and response to mitigate the impact of these psychological outcomes [7]. Yet the cross-sectional design of this study precludes any inference about temporal or causal pathways. Accordingly, longitudinal research is needed in this setting to clarify developmental trajectories, determine whether the BPFSC-11 can detect changes over time, establish its suitability for monitoring intervention outcomes or informing policy initiatives. Once confirmed, together with the CESDR-V, these two instruments may help inform future efforts to enhance social service delivery and guide psychosocial support initiatives, though any application to policy or system-level reforms would require substantial longitudinal and clinical validation.

### Strengths and limitations

This study benefits from a large school-based sample, rigorous translation and cultural adaptation procedures, validated measures of CM and depression, and the application of multi-group CFA to test gender invariance. However, several limitations should be noted. First, the cross-sectional design prevents conclusions about causality or developmental trajectories. Further research is needed to explore how different types and severities of maltreatment may be differentially related to these outcomes. Second, although school attendance is high at 95.6% in Vietnam [45], adolescents who are out of school, who may experience greater adversity, were not captured. Thus, the study might underestimate BPF and maltreatment, and the psychometric properties of the BPFSC-11 might differ in more marginalised adolescent populations. The third limitation is the reliance on self-report measures and the absence of a back-translation step, although the rigorous cultural adaptation procedures employed help mitigate, though not entirely eliminate, this concern. Fourth, although the unidimensional model fit well, competing multidimensional models were not tested; future work should evaluate alternative structures to ensure that the single-factor solution is not sample-specific. Fifth, the strong correlations between BPF and depressive symptoms suggest potential overlap with general internalising distress, and clinical assessments remain necessary to differentiate BPF. Sixth, the absence of test–retest reliability limits conclusions about temporal stability. Finally, this study is not nationally representative, as participants were drawn from a single province in Northern Vietnam. Although the province is broadly comparable to national averages on key socioeconomic and health indicators [45], this similarity can only partially mitigate the concerns regarding representativeness.

### Future directions

Future longitudinal research is needed to examine developmental trajectories of BPF and the effects of CM on later mental health outcomes. The measures of CM are necessarily retrospective because CM carries the ethical imperative to act to protect the child’s safety. Longitudinal studies may also provide evidence on how mental health problems compound over time and which factors may strengthen the resilience and recovery of children previously exposed to CM. Clinical validation studies are also essential to determine the discriminant validity and diagnostic utility of the BPFSC-11. Longitudinal invariance and broader measurement invariance testing across age groups, regions, socioeconomic strata, and ultimately across countries will also strengthen the evidence base for the scale’s cross-cultural applicability.

## Conclusions

The study provides support for the validity and reliability of the BPFSC-11 in measuring BPF among Vietnamese adolescents. With only 11 clearly worded items, the scale is brief, easy to administer, and potentially cost-effective for use in school and community settings in Vietnam, and it may also hold value for other low-resource contexts. When used alongside the CESDR-V, the BPFSC-11 could contribute to the monitoring of adolescents’ psychosocial well-being, and serve as a robust indicator of maltreatment-related affective and personality disturbance in non-clinical settings, as well as a potential outcome measure in child protection and intervention studies. The present findings represent an important first step, but further validation across diverse regions of Vietnam and within clinical samples is necessary before the BPFSC-11 can be considered suitable for diagnostic screening. Future longitudinal research is also warranted to clarify the directionality of associations between CM, BPF, and depression, and to better understand developmental trajectories over time.

## Data Availability

The data that support the findings of this study are available under the larger project entitled “The Role of Schools in Detecting and Responding to Child Maltreatment: A Collaborative Approach to Evidence-based Policy Development in Switzerland and Vietnam” (code IZVSZ1.203300) but restrictions apply to the availability of these data, which were used under license for the current study, and so are not publicly available. Data are, however, available from the corresponding author upon reasonable request and with permission of the lead investigator of the aforementioned project.

## Abbreviations

BPF: Borderline personality features
BPFSC-11: Borderline Personality Features Scale for Children
BPD: Borderline personality disorder
CAMI: Computer-Assisted Maltreatment Inventory scale
CES-D: Centre for Epidemiological Survey Depression Scale
CESDR-V: Centre for Epidemiological Survey Depression Scale – Vietnamese version
CFA: Confirmatory factor analysis
CM: Child maltreatment
DASS-21: Depression, Anxiety and Stress Scales 21 items
EFA: Exploratory factor analysis
IPV: Intimate partner violence
MSI-BPD: McLean Screening Instrument for Borderline Personality Disorder

## References

[1] Agarwal N, Kumar Jha A. Adolescence. In: Shackelford T, editor. Encyclopedia of Religious Psychology and Behavior. Cham: Springer Nature Switzerland; 2025. pp. 1–11.

[2] American Psychiatric Association. Diagnostic and Statistical Manual of Mental Disorders. DSM-5-TR. 5th edition, text revision. ed. Washington, DC: American Psychiatric Association Publishing; 2022.

[3] Gunderson JG, Hoffman PD. Understanding and treating borderline personality disorder: A guide for professionals and families. 1st ed. Washington, DC: American Psychiatric Pub.; 2005.

[4] Chanen AM, Kaess M. Developmental pathways to borderline personality disorder. Curr Psychiatry Rep. 2012;14(1):45–53. 22009682 10.1007/s11920-011-0242-y

[5] Salavati M, Selby EA. Childhood maltreatment and borderline personality disorder. In: Salavati M, Selby EA, editors. Theories of Borderline Personality Disorder: Concepts and Empirical Base. Cham: Springer Nature Switzerland; 2024. pp. 57–94.

[6] Leichsenring F, Fonagy P, Heim N, Kernberg OF, Leweke F, Luyten P, et al. Borderline personality disorder: A comprehensive review of diagnosis and clinical presentation, etiology, treatment, and current controversies. World Psychiatry. 2024;23(1):4–25. 38214629 10.1002/wps.21156 PMC10786009

[7] Sharp C, Steinberg L, Temple J, Newlin E. An 11-Item measure to assess borderline traits in adolescents: Refinement of the BPFSC using IRT. Pers Disord. 2014;5(1):70–8. 10.1037/per0000057 PMC10754293 24588063

[8] Crick NR, Murray-Close D, Woods K. Borderline personality features in childhood: A short-term longitudinal study. Dev Psychopathol. 2005;17(4):1051–70. 16613430

[9] Allman M, Barkauskienė R, Gervinskaitė-Paulaitienė L, Sharp C. Evaluation of the borderline personality features scale for children and life satisfaction in lithuanian adolescents. Eur J Psychol Assess. 2025.

[10] Aune T, Peterson R, Aune SF. Psychometric properties and measurement invariance of the borderline personality features scale for children (BPFSC-11). Psychol Rep. 2025:332941251358207. 10.1177/00332941251358207 40605779

[11] Bibi H, Kazmi SF. Urdu translation and validation of 11-Item measure to assess borderline personality features in Pakistani adolescents. SAGE Open. 2021;11(1):2158244020986157.

[12] Calvo N, Marin JL, Vidal R, Sharp C, Duque JD, Ramos-Quiroga J-A, et al. Discrimination of Borderline Personality Disorder (BPD) and Attention-Deficit/Hyperactivity Disorder (ADHD) in adolescents: Spanish version of the Borderline Personality Features Scale for Children-11 self-Report (BPFSC-11) Preliminary results. Borderline Personal Disord Emot Dysregul. 2023;10(1):15–1. 37189168 10.1186/s40479-023-00223-2 PMC10185374

[13] Carreiras D, Loureiro M, Cunha M, Sharp C, Castilho P. Validation of the Borderline Personality Features Scale for Children (BPFS-C) and for Parents (BPFS-P) for the Portuguese Population. J Child Fam Stud. 2020;29(11):3265–75.

[14] Ensink K, Bégin M, Kotiuga J, Sharp C, Normandin L. Psychometric properties of the French version of the Borderline Personality Features Scale for children and adolescents. Adolesc Psychiatry. 2020;10(1):48–58.

[15] Fossati A, Sharp C, Borroni S, Somma A. Psychometric properties of the Borderline Personality Features Scale for Children-11 (BPFSC-11) in a sample of community dwelling Italian adolescents. Eur J Psychol Assess. 2019;35(1):70–7.

[16] Vanwoerden S, Garey L, Ferguson T, Temple JR, Sharp C. Borderline Personality Features Scale for Children-11: Measurement invariance over time and across gender in a community sample of adolescents. Psychol Assess. 2019;31(1):114–9. 30080065 10.1037/pas0000640 PMC6312464

[17] Haltigan JD, Vaillancourt T, The Borderline Personality Features Scale for Children (BPFS-C). Factor structure and measurement invariance across time and sex in a community-based sample. J Psychopathol Behav Assess. 2016;38(4):600–14.

[18] Mathews B, Pacella R, Dunne MP, Simunovic M, Marston C. Improving measurement of child abuse and neglect: A systematic review and analysis of national prevalence studies. PLoS ONE. 2020;15(1):e0227884. 31990913 10.1371/journal.pone.0227884 PMC6986759

[19] World Health Organization. Child maltreatment. https://www.who.int/news-room/fact-sheets/detail/child-maltreatment. Accessed 24 Aug 2025.

[20] Gardner MJ, Thomas HJ, Erskine HE. The association between five forms of child maltreatment and depressive and anxiety disorders: A systematic review and meta-analysis. Child Abuse Negl. 2019;96:104082. 31374447 10.1016/j.chiabu.2019.104082

[21] Hoang TNM. Borderline features in vietnamese adolescence: the roles of childhood trauma, parental bonding, and family functioning [Doctoral dissertation]. US: University of Minnesota; 2014.

[22] Ibrahim J, Cosgrave N, Woolgar M. Childhood maltreatment and its link to borderline personality disorder features in children: A systematic review approach. Clin Child Psychol Psychiatry. 2018;23(1):57–76. 28617046 10.1177/1359104517712778

[23] Sharp C, Vanwoerden S, Jouriles EN, Godfrey DA, Babcock J, McLaren V, et al. Exposure to interparental intimate partner violence and the development of borderline features in adolescents. Child Abuse Negl. 2020;103:104448. 32171797 10.1016/j.chiabu.2020.104448 PMC10176899

[24] Linehan M. Cognitive-behavioral treatment of borderline personality disorder. New York: Guilford Press; 1993.

[25] Benczkowski T. The relationship between adverse childhood experiences and borderline personality disorder among inpatient adolescents [Doctoral dissertation]. US: William James College; 2023.

[26] Marchetti D, Musso P, Verrocchio MC, Manna G, Kopala-Sibley DC, De Berardis D, et al. Childhood maltreatment, personality vulnerability profiles, and borderline personality disorder symptoms in adolescents. Dev Psychopathol. 2022;34(3):1163–76. 33494855 10.1017/S0954579420002151

[27] Porter C, Palmier-Claus J, Branitsky A, Mansell W, Warwick H, Varese F. Childhood adversity and borderline personality disorder: a meta-analysis. Acta Psychiatr Scand. 2020;141(1):6–20. 31630389 10.1111/acps.13118

[28] Liu Y, Li B, Hao F, Wang B, Zhu J, Liu D, et al. Associations between borderline personality disorder features and the risk of first onset major depressive disorder: Findings from a 2-year longitudinal study in a sample of first-year university students in China. J Affect Disord. 2021;295:5–10. 34385011 10.1016/j.jad.2021.08.008

[29] Yen S, Peters JR, Nishar S, Grilo CM, Sanislow CA, Shea MT, et al. Association of borderline personality disorder criteria with suicide attempts: Findings from the collaborative longitudinal study of personality disorders over 10 years of follow-up. JAMA Psychiatry. 2021;78(2):187–94. 33206138 10.1001/jamapsychiatry.2020.3598 PMC7675214

[30] Bach B, Fjeldsted R. The role of DSM-5 borderline personality symptomatology and traits in the link between childhood trauma and suicidal risk in psychiatric patients. Borderline Personal Disord Emot Dysregul. 2017;4:12. 28638621 10.1186/s40479-017-0063-7 PMC5474295

[31] Lin J, Zou L, Lin W, Becker B, Yeung A, Cuijpers P, et al. Does gender role explain a high risk of depression? A meta-analytic review of 40 years of evidence. J Affect Disord. 2021;294:261–78. 34304081 10.1016/j.jad.2021.07.018

[32] Ferrari AJ, Somerville AJ, Baxter AJ, Norman R, Patten SB, Vos T, et al. Global variation in the prevalence and incidence of major depressive disorder: A systematic review of the epidemiological literature. Psychol Med. 2013;43(3):471–81. 22831756 10.1017/S0033291712001511

[33] Qian X, Townsend ML, Tan WJ, Grenyer BFS. Sex differences in borderline personality disorder: A scoping review. PLoS ONE. 2022;17(12):e0279015. 36584029 10.1371/journal.pone.0279015 PMC9803119

[34] PopulationPyramid.net. Population Pyramid of Vietnam. 2025. https://www.populationpyramid.net/viet-nam/2025/. Accessed 23 March 2025.

[35] Le MTH, Nguyen HT, Tran TD, Fisher JRW. Experience of low mood and suicidal behaviors among adolescents in Vietnam: Findings from two national population-based surveys. J Adolesc Health. 2012;51(4):339–48. 22999834 10.1016/j.jadohealth.2011.12.027

[36] Tran QA, Le VTH, Nguyen THD. Depressive symptoms and suicidal ideation among Vietnamese students aged 13–17: Results from a cross-sectional study throughout four geographical regions of Vietnam. Health Psychol Open. 2020;7(2):2055102920973253. 33240521 10.1177/2055102920973253 PMC7672740

[37] Tang JJ, Yu Y, Wilcox HC, Kang C, Wang K, Wang C, et al. Global risks of suicidal behaviours and being bullied and their association in adolescents: School-based health survey in 83 countries. EClinicalMedicine. 2020;19:100253. 32140671 10.1016/j.eclinm.2019.100253 PMC7046520

[38] Thai TT, Jones MK, Nguyen TP, Pham TV, Bui HHT, Kim LX, et al. The prevalence, correlates and functions of non-suicidal self-injury in Vietnamese adolescents. Psychol Res Behav Manag. 2021;14:1915–27. 34866944 10.2147/PRBM.S339168 PMC8636691

[39] Tran NK, Alink LRA, Van Berkel SR, Van Ijzendoorn MH. Child maltreatment in Vietnam: Prevalence and cross-cultural comparison. Aggress Maltreat Trauma. 2017;26(3):211–30.

[40] Nguyen HT, Dunne MP, Le AV. Multiple types of child maltreatment and adolescent mental health in Viet Nam. Bull World Health Organ. 2010;88(1):22–30. 20428350 10.2471/BLT.08.060061 PMC2802435

[41] Pham TS, Qi H, Chen D, Chen H, Fan F. Prevalences of and correlations between childhood trauma and depressive symptoms, anxiety symptoms, and suicidal behavior among institutionalized adolescents in Vietnam. Child Abuse Negl. 2021;115:105022. 33677169 10.1016/j.chiabu.2021.105022

[42] Thai TT, Cao PLT, Kim LX, Tran DP, Bui MB, Bui HHT. The effect of adverse childhood experiences on depression, psychological distress and suicidal thought in Vietnamese adolescents: Findings from multiple cross-sectional studies. Asian J Psychiatr. 2020;53:102134. 32447255 10.1016/j.ajp.2020.102134

[43] Radloff LS, The CES-D, Scale. A self-report depression scale for research in the general population. Appl Psychol Meas. 1977;1(3):385–401.

[44] Murphy J, Goldner EM, Goldsmith CH, Oanh PT, Zhu W, Corbett KK, et al. Selection of depression measures for use among Vietnamese populations in primary care settings: A scoping review. Int J Ment Health Syst. 2015;9(1):31. 26300962 10.1186/s13033-015-0024-8 PMC4543473

[45] General Statistics Office. Statistical yearbook of Vietnam 2023. Statistical Publishing House. 2023. https://www.nso.gov.vn/en/default/2024/07/statistical-yearbook-of-2023/. Accessed 24 Jan 2025.

[46] Le MT, Nguyen T, Dinh TH, Bui TP, Nguyen TH. Application of digital survey tools to screen for child maltreatment among secondary school students in Vietnam. The First International Conference on Responsible Artificial Intelligence and Data Science. EAI/Springer innovations in communication and computing: Springer; 2024.

[47] Tran T, Nguyen H, Shochet I, Nguyen N, La N, Wurfl A, et al. Psychometric properties of the Centre for Epidemiologic Studies Depression Scale Revised – Vietnamese version (CESDR-V) among adolescents. Psychiatry Res Commun. 2024;4(2):100165.

[48] Nguyen T. Trai nghiem nguoc dai va moi lien quan voi tram cam: Ket qua dieu tra o học sinh tại 08 truong trung học co so tinh Bac Giang nam 2023 (Experiences of maltreatment and their association with depression: Findings from a survey of students at eight lower secondary schools in Bac Giang province) [Master dissertation]. Vietnam: Hanoi University of Public Health; 2024.

[49] DiLillo D, Hayes-Skelton SA, Fortier MA, Perry AR, Evans SE, Messman Moore TL, et al. Development and initial psychometric properties of the Computer Assisted Maltreatment Inventory (CAMI): A comprehensive self-report measure of child maltreatment history. Child Abuse Negl. 2010;34(5):305–17. 20347148 10.1016/j.chiabu.2009.09.015

[50] Bennett K, Heritage B, Allen P. SPSS statistics: A practical guide. 5th edition. ed. South Melbourne, Victoria: Cengage Learning Australia; 2023.

[51] Mai R, Niemand T, Kraus S. A tailored-fit model evaluation strategy for better decisions about structural equation models. Technol Forecast Soc Chang. 2021;173:121142.

[52] Chen FF. Sensitivity of goodness of fit indexes to lack of measurement invariance. Struct Equ Model. 2007;14(3):464–504.

[53] IBM Corp. IBM SPSS Statistics for Windows (Version 30.0) [Computer software]. IBM. 2024. https://www.ibm.com/analytics/spss-statistics-software

[54] The Jamovi Project. Jamovi (Version 2.6.44) [Computer software]. 2025. https://www.jamovi.org

[55] Humphreys KL, LeMoult J, Wear JG, Piersiak HA, Lee A, Gotlib IH. Child maltreatment and depression: A meta-analysis of studies using the Childhood Trauma Questionnaire. Child Abuse Negl. 2020;102:104361. 32062423 10.1016/j.chiabu.2020.104361 PMC7081433

[56] Li M, Gao T, Su Y, Zhang Y, Yang G, D’Arcy C, et al. The timing effect of childhood maltreatment in depression: A systematic review and meta-analysis. Trauma Violence Abuse. 2023;24(4):2560–80. 35608502 10.1177/15248380221102558

[57] Mehta, Kelly AB, Laurens KR, Haslam D, Williams KE, Walsh K, et al. Child maltreatment and long-term physical and mental health outcomes: An exploration of biopsychosocial determinants and implications for prevention. Child Psychiatry Hum Dev. 2023;54(2):421–35. 34586552 10.1007/s10578-021-01258-8 PMC8480117

[58] An TL, Waling A, Bourne A. Men and masculinities studies in Vietnam: A brief review. Sociol Compass. 2022;16(3):e12965.

[59] Ji K, Finkelhor D. A meta-analysis of child physical abuse prevalence in China. Child Abuse Negl. 2015;43:61–72. 25498804 10.1016/j.chiabu.2014.11.011

[60] Spence N, Lan NTT. Family sustainability and child protection in Vietnam. Child Youth Serv Rev. 2021;122:105884.

[61] Fontes LA, Plummer C. Cultural issues in disclosures of child sexual abuse. J Child Sex Abus. 2010;19(5):491–518. 20924908 10.1080/10538712.2010.512520

[62] Merkin RS. Individualism-Collectivism and saving face. In: Merkin RS, editor. Saving Face in Business. New York: Palgrave Macmillan US; 2018. pp. 81–117.

[63] Vo LH. Conceptualising social categories in vietnamese interpersonal relationships: a folk perspective. Int J Innov Interdiscip Res. 2015;3(1).

[64] Chanen AM, Jovev M, McCutcheon LK, Jackson HJ, McGorry PD. Borderline personality disorder in young people and the prospects for prevention and early intervention. Curr Psychiatry Rev. 2008;4(1):48–57.

[65] UNICEF. Adolescent data portal. https://data.unicef.org/adp/country/vnm/. Accessed 15 July 2025.

